# Urethrogram-Directed Stereotactic Body Radiation Therapy for Clinically Localized Prostate Cancer in Patients with Contraindications to Magnetic Resonance Imaging

**DOI:** 10.3389/fonc.2015.00194

**Published:** 2015-09-01

**Authors:** Ima Paydar, Brian S. Kim, Robyn A. Cyr, Harriss Rashid, Amna Anjum, Thomas M. Yung, Siyuan Lei, Brian T. Collins, Simeng Suy, Anatoly Dritschilo, John H. Lynch, Sean P. Collins

**Affiliations:** ^1^Department of Radiation Medicine, Georgetown University Hospital, Washington, DC, USA; ^2^Department of Urology, Georgetown University Hospital, Washington, DC, USA

**Keywords:** prostate cancer, SBRT, urethrogram, CyberKnife, magnetic resonance imaging

## Abstract

**Purpose:**

Magnetic resonance imaging (MRI)-directed stereotactic body radiation therapy (SBRT) has been established as a safe and effective treatment for prostate cancer. For patients with contraindications to MRI, CT-urethrogram is an alternative imaging approach to identify the location of the prostatic apex to guide treatment. This study sought to evaluate the safety of urethrogram-directed SBRT for prostate cancer.

**Methods:**

Between February 2009 and January 2014, 31 men with clinically localized prostate cancer were treated definitively with urethrogram-directed SBRT with or without supplemental intensity-modulated radiation therapy (IMRT) at Georgetown University Hospital. SBRT was delivered either as a primary treatment of 35–36.25 Gy in five fractions or as a boost of 19.5 Gy in three fractions followed by supplemental conventionally fractionated IMRT (45–50.4 Gy). Toxicities were recorded and scored using the Common Terminology Criteria for Adverse Events version 4.0 (CTCAE v.4.0).

**Results:**

The median patient age was 70 years with a median prostate volume of 38 cc. The median follow-up was 3.7 years. The patients were elderly (Median age = 70), and comorbidities were common (Carlson comorbidity index ≥2 in 36%). Seventy-one percent of patients utilized alpha agonists prior to treatment, and 9.7% had prior procedures for benign prostatic hyperplasia. The 3-year actuarial incidence rates of ≥Grade 3 GU toxicity and ≥Grade 2 GI toxicity were 3.2 and 9.7%, respectively, and there were no Grade 4 or 5 toxicities.

**Conclusion:**

Magnetic resonance imaging is the preferred imaging modality to guide prostate SBRT treatment. However, urethrogram-directed SBRT is a safe alternative for the treatment of patients with prostate cancer who are unable to undergo MRI.

## Introduction

Stereotactic body radiation therapy (SBRT) treats prostate cancer with large doses of radiation per fraction (6.5–9 Gy) to take advantage of the postulated radiobiological model of improved tumor cell kill with decreased normal tissue toxicity (1, 2). It may be used as a primary treatment for favorable prostate cancer (3, 4) or as a boost to conventional pelvic radiation therapy in unfavorable prostate cancer (5, 6). Early clinical results suggest that these approaches provide high rates of biochemical control with acceptable toxicity (7–9). Based on the increased convenience of an abbreviated treatment, SBRT usage is likely to increase.

The goal of prostate SBRT is to treat the entire prostate and proximal seminal vesicles while limiting radiation dose to the adjacent critical structures, including the bladder, rectum, and membranous urethra. Since the prostate apex is commonly involved with cancer, under-dosing this region would likely increase the risk of recurrence (10). Also, identification of the membranous urethra remains critical during treatment planning as it is the most common location for radiation therapy-induced strictures (11). However, poor soft tissue resolution of conventional CT scans prevents adequate visualization of the transition between the prostate apex and membranous urethra (12–14). Moreover, the utilization of bony and soft tissue anatomical landmarks to identify these critical structures is difficult and prone to error and inter-user variability (15, 16). Reliance on a CT scan alone, therefore, poses the risk of decreased dose to the prostate apex or increased dose to the membranous urethra. Such an uncertainty in treatment planning becomes a heightened concern because the large radiation doses and steep dose gradients characteristic of SBRT – when incorrectly administered – may result in a high rate of recurrence or urethral stricture.

The standard approach to treatment planning, therefore, utilizes non-invasive magnetic resonance (MR) imaging to delineate adjacent critical structures, such as the bladder neck, rectum, and membranous urethra (12, 13). MR imaging better defines the prostate and reduces the overall target volume by 30% relative to CT imaging (17, 18). In addition, the prostatic-rectal and prostatic-bladder interfaces are better defined by MR than by CT imaging. The membranous urethra, which varies in length, is clearly visible on a magnetic resonance imaging (MRI). MR imaging also better defines the prostatic apex, which allows for dose reduction to the genitourinary diaphragm (GUD).

A dilemma arises when patients with a contraindication to MR imaging, such as presence of a pacemaker, defibrillator, or metallic foreign bodies, present for radiation treatment (19, 20). For these patients, an alternative imaging modality must be employed. Urethrograms have previously been used to identify the distal extent of the membranous urethra and the approximate location of the prostate apex for the treatment of prostate cancer with conventional radiation therapy (21–23). In these studies, the distance of the membranous urethra from the GUD (beak of the urethrogram) to the prostate apex is approximately 1.0–1.5 cm (23–25), though the distance can significantly vary between patients (range, 0.5–2.0 cm) (13, 26, 27). While a urethrogram does not locate the prostate apex directly, an identifiable point that is anterior and inferior to the prostate can be used to infer the relative location of the prostate. To the best of our knowledge, no studies on urethrogram-directed SBRT for prostate cancer have been published. Herein, we report our toxicity outcomes following urethogram-directed SBRT for clinically localized prostate cancer.

## Materials and Methods

### Patient selection

Patients eligible for study inclusion had prostate cancer treated with urethrogram-directed SBRT. Patients treated with MRI-directed SBRT were excluded. Prospectively collected toxicity data for all patients included in our institutional database were analyzed with Internal Review Board (IRB) approval. Relative comorbidity was assessed using the Charlson comorbidity index (CCI), as previously described (28) with higher scores representing increased severity.

### SBRT treatment planning and delivery

**Figure 1:**
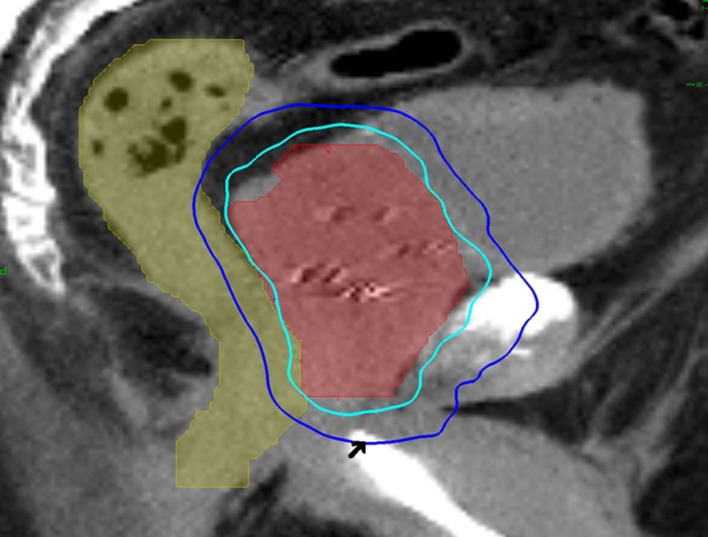
**A 60-year-old man with intermediate risk prostate cancer had cardiac disease and an internal defibrillator, which precluded magnetic resonance imaging for treatment planning**. Thus, he was treated with urethrogram-directed SBRT: treatment planning sagittal computed tomography urethrogram images demonstrating the prostate (red) and rectum (green) are shown. Arrowhead marks the “beak” of the urethrogram. Isodose lines shown as follows: 100% of the prescription dose (light blue line) and 50% of the prescription dose (dark blue line).

Stereotactic body radiation therapy treatment planning and delivery were conducted, as previously published with minor modifications (3, 29). Four to six gold markers were placed into the prostate transrectally or under transrectal ultrasound guidance. Seven days after fiducial placement, patients underwent a treatment planning CT scan with a retrograde urethrogram (21). The patient was placed in a supine position. To conduct the urethrogram, approximately 8 cc of contrast was injected into the penile uretrhra and a penile clamp was applied (22, 23). Next, a CT scan using 1.25 mm axial slices was completed. The beak of the urethrogram was defined as the most superior CT slice with contrast in the urethra. The clinical target volume (CTV), including the prostate and the proximal seminal vesicles, was defined utilizing this beak as well as other anatomical landmarks (15, 16). The CTV was expanded 3 mm posteriorly and 5 mm in all other dimensions to create the PTV. The bladder and membranous urethra were contoured and evaluated with dose–volume histogram (DVH) analysis during treatment planning using Multiplan (Accuray Inc., Sunnyvale, CA, USA) inverse treatment planning technique, as previously described (3, 5). Target position was verified multiple times during each treatment using paired, orthogonal x-ray images (30). An example of a treatment plan is provided in Figure 1.

### Follow-up and statistical analysis

Pre-treatment function was documented before treatment (31–33), and post-treatment toxicity was prospectively recorded during routine follow-up visits 1 month after the completion of radiation treatment, every 3 months for the first year, every 6 months for the second and third years, then yearly (3). Toxicity was documented at follow-up visits using the National Cancer Institute (NCI) Common Toxicity Criteria for Adverse Events (CTCAE) version 4.0. Acute toxicity was defined as toxicity experienced during or within 6 months of radiation therapy. Late toxicity was defined as occurring at least 6 months after delivery of radiation therapy. In general, Grade 2 toxicity is defined as symptoms requiring medication (i.e., alpha-antagonist or antidiarrheal medications) or laser coagulation. Grade 3 toxicity indicates complications requiring minor surgical intervention (i.e., urethral dilation). Actuarial likelihood estimates for toxicities were determined using the Kaplan–Meier method.

## Results

**Figure 2:**
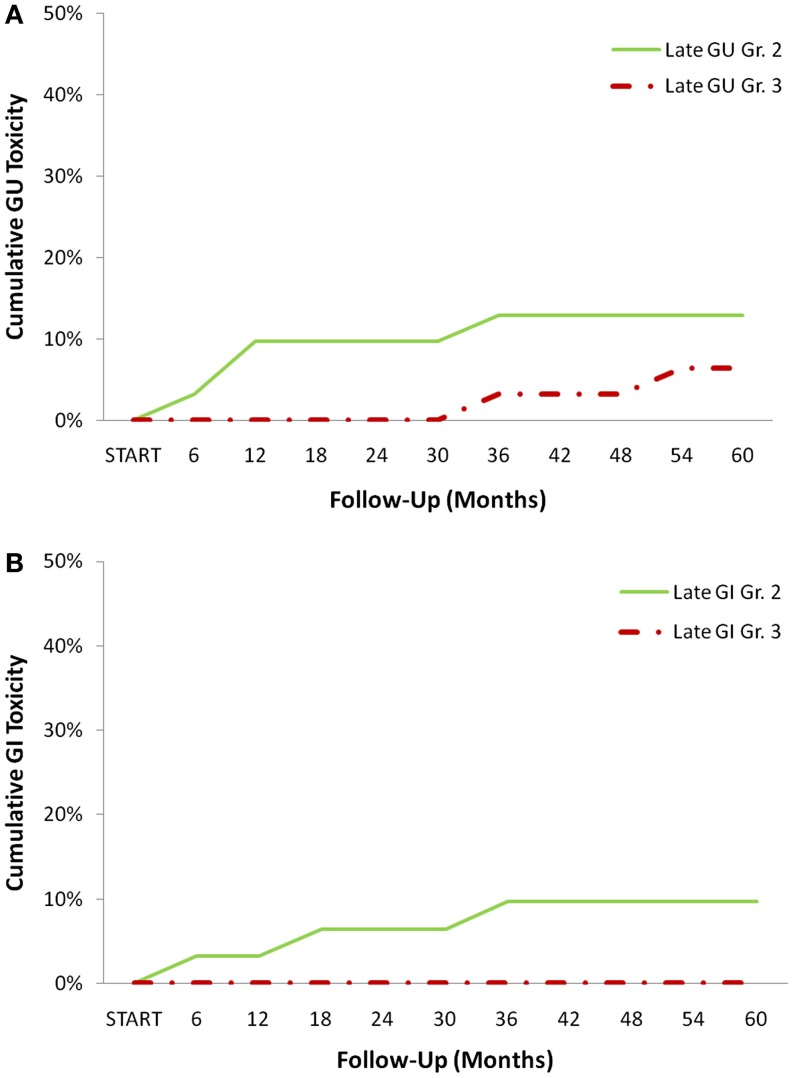
**Cumulative late CTCAE graded toxicities: (A) genitourinary (GU) and (B) Gastrointestinal (GI) toxicities at each time point**.

From February 2009 to January 2014, 31 prostate cancer patients were treated with urethrogram-directed SBRT (Table 1). The median follow-up was 3.7 years. Patients were ethnically diverse, with 54.8% of non-Caucasian ancestry. Median age was 70 years (range, 56–85 years). The most common contraindication to MRI was a pacemaker/defibrillator (58%). Comorbidities were common (CCI ≥2 in 36%). Fifty-five percent of patients had moderate to severe lower urinary tract symptoms prior to treatment (baseline AUA ≥8) with a median baseline AUA of 8 (Table 2). The median prostate volume was 38 (13–75) cc, and 9.7% had prior procedures for benign prostatic hyperplasia (BPH). Seventy-one percent of patients utilized alpha-antagonists prior to SBRT. By D’Amico classification, 3 patients had low-, 19 intermediate-, and 9 high-risk diseases. Eleven patients (35.5%) also received androgen deprivation therapy (ADT). Seventy-five percent of the patients were treated with 35–36.25 Gy in five fractions. The remaining 25% of the patients were treated with 19.5 Gy in three 6.5 Gy fractions delivered via SBRT and had supplemental radiation delivered using intensity-modulated radiation therapy (IMRT) (median of 45 Gy over 25 fractions). There was a biochemical failure in one intermediate risk patient. At last follow-up, 26 (84%) were alive, and 5 (16%) had died from non-prostate cancer causes. Actuarial incidence rates of late GU and GI toxicities are demonstrated in Figure 2. The 3-year actuarial incidence rates of ≥Grade 3 GU toxicity and ≥Grade 2 GI toxicity were 3.2% and 9.7%, respectively, and there were no Grade 4 or 5 toxicities.

**Table 1:** **Baseline patient characteristics and treatment**.

		Patients	*n*
		*N* ***=*** 31	
Age (years)	Median 70 (56–85)		
	<60	6.5%	2
	60–69	41.9%	13
	70–79	41.9%	13
	>80	9.7%	3
Race	White	45.2%	14
	Black	45.2%	14
	Other	9.6%	3
Charleson comorbidity index	CCI = 0	25.8%	8
	CCI = 1	38.7%	12
	CCI > 2	35.5%	11
Contraindication to MRI	Pacemaker/defibrillator	58.1%	18
	Metal	41.9%	13
Prostate volume (cc)	Median 38 (13–75)		
Pre-txt PSA (ng/ml)	Median 6.5 (0.8–148)		
	<10	67.7%	21
	>10 and <20	12.9%	4
	>20	19.4%	6
T stage	Tib	3.2%	1
	Tic	67.8%	21
	T2a	3.2%	1
	T2b	12.9%	4
	T2c	12.9%	4
Gleason score	6 (3 + 3)	16.1%	5
	7 (3 + 4; 4 + 3)	61.3%	19
	8 (4 + 4)	9.7%	3
	9 (4 + 5; 5 + 4)	12.9%	4
Risk groups (D’Amico)	Low	9.7%	3
	Intermediate	61.3%	19
	High	29.0%	9
Hormone treatment	Yes	35.5%	11
	No	64.5%	20
Anti-coagulant use	Yes	19.4%	25
	No	80.6%	6

**Table 2:** **Pre-treatment quality of life (QOL) scores**.

	% Patients (*n* ***=*** 31)		
**Baseline AUA score**			
0–7 (Mild)		45.2%	
8–19 (Moderate)		41.9%	
>20 (Severe)		12.9%	

	**Mean**	**SD**	**MID**

**Baseline EPIC-26 summary score**
Urinary domain	82.7	17.8	8.9
Incontinence domain	85.6	22.1	11.1
Irritative/obstructive domain	83.5	16.0	8.0
Bowel domain	94.0	9.4	4.7
**Baseline EPIC-26 bother score**			
Urinary domain	68.3	30.7	15.4
Bowel domain	85.8	24.3	12.1
**Baseline SF-12 score**			
PCS	45.6	10.2	5.1
MCS	53.8	8.3	4.2

## Discussion

To the best of our knowledge, this is the first report on urethrogram-directed prostate SBRT. In this study, the rates of Grade 2 and higher toxicities were higher than that previously published for prostate SBRT (3–7). The etiology of this increased toxicity is unclear but likely multifactorial. For example, patients with high comorbidity scores have been shown to be at increased risk of radiation therapy-related toxicity (34). The patients included in this study were elderly with a high level of comorbidity prior to treatment. Specifically, 33% of patients had >2 comorbid conditions, and 19% utilized anticoagulants. Another likely reason is the increased uncertainty in location of the membranous urethra and anterior rectal wall with respect to the prostate when using urethrogram-based treatment planning. As expected, this is likely due to the poor visualization of soft tissue structures. Yet, significant toxicity was acceptable in our patient population. There were two bulbar urethral strictures that were managed with dilation and did not recur. Three patients experienced late rectal bleeding that resolved with coagulation. All three patients were taking anticoagulants at the time of rectal bleeding.

This study had several limitations. First, the number of patients treated was small as contraindications to MR imaging were rare at our institution. Our results may not be generalizable to the overall patient population since the high rate of comorbidites in these patients may have rendered them more prone to toxicities. Also, pre-treatment cystoscopies were not performed due to the risk of iatrogenic strictures. Therefore, there may have been a higher than expected baseline rate of subclinical urethral strictures in this patient population that remained undetected until follow-up. Finally, long-term follow-up will be necessary to confirm our reported late urinary effects.

## Conclusion

Magnetic resonance imaging is the preferred imaging modality to guide SBRT treatment. However, for patients with a contraindication to MRI, urethrogram-directed SBRT is a safe alternative for the treatment of localized prostate cancer. Long-term follow-up with more patients will be necessary to confirm these findings.

## Conflict of Interest Statement

Sean P. Collins and Brian T. Collins serve as clinical consultants to Accuray Inc. The Department of Radiation Medicine at Georgetown University Hospital receives a grant from Accuray to support a research coordinator. The other authors declare that they have no competing interests.

## Abbreviations

ADT, androgen deprivation therapy; AUA, American Urological Association; BED, biologically effective dose; BPH, benign prostatic hyperplasia; CCI, Charlson comorbidity index; CT, computed tomography; CTV, clinical target volume; DVH, dose–volume histogram; EQD2, equivalent dose in 2-Gy fractions; EPIC, expanded prostate index composite; GTV, gross target volume; Gy, Gray; IMRT, intensity-modulated radiation therapy; IRB, Internal Review Board; MCS, Mental Health Composite Score; MR, magnetic resonance; PCS, Physical Health Composite Score; PTV, planning target volume, SD, standard deviation; SF, 12, Short Form, 12 Health Survey; SBRT, stereotactic body radiation therapy.
